# Bu Shen Yi Sui Capsule Alleviates Neuroinflammation and Demyelination by Promoting Microglia toward M2 Polarization, Which Correlates with Changes in miR-124 and miR-155 in Experimental Autoimmune Encephalomyelitis

**DOI:** 10.1155/2021/5521503

**Published:** 2021-03-16

**Authors:** Zheng Zha, Yan-Fang Gao, Jing Ji, Ya-Qin Sun, Jun-Ling Li, Fang Qi, Nan Zhang, Liang-Yun Jin, Bing Xue, Tao Yang, Yong-Ping Fan, Hui Zhao, Lei Wang

**Affiliations:** ^1^School of Traditional Chinese Medicine, Beijing Key Lab of TCM Collateral Disease Theory Research, Capital Medical University, Beijing 100069, China; ^2^Core Facility Center, Capital Medical University, Beijing 100069, China; ^3^Beijing Tian Tan Hospital, Capital Medical University, Beijing 100070, China

## Abstract

**Background:**

Bu Shen Yi Sui capsule (BSYS) is a traditional Chinese medicine prescription that has shown antineuroinflammatory and neuroprotective effects in treating multiple sclerosis (MS) and its animal model of experimental autoimmune encephalomyelitis (EAE). Microglia play an important role in neuroinflammation. The M1 phenotype of microglia is involved in the proinflammatory process of the disease, while the M2 phenotype plays an anti-inflammatory role. Promoting the polarization of microglia to M2 in MS/EAE is a promising therapeutic strategy. This study is aimed at exploring the effects of BSYS on microglial polarization in mice with EAE.

**Methods:**

The EAE model was established by the intraperitoneal injection of pertussis toxin and subcutaneous injection of myelin oligodendrocyte glycoprotein (MOG)_35-55_ in C57BL/6J mice. The mice were treated with BSYS (3.02 g/kg), FTY720 (0.3 mg/kg), or distilled water by intragastric administration. H&E and LFB staining, transmission electron microscopy, qRT-PCR, immunofluorescence, ELISA, fluorescence in situ hybridization, and western blotting were used to detect the histological changes in myelin, microglial M1/M2 polarization markers, and the expression of key genes involved in EAE. *Results and Conclusions*. BSYS treatment of EAE mice increased the body weight, decreased the clinical score, and reduced demyelination induced by inflammatory infiltration. BSYS also inhibited the mRNA expression of M1 microglial markers while increasing the mRNA level of M2 markers. Additionally, BSYS led to a marked decrease in the ratio of M1 microglia (iNOS^+^/Iba1^+^) and an obvious increase in the number of M2 microglia (Arg1^+^/Iba1^+^). In the EAE mouse model, miR-124 expression was decreased, and miR-155 expression was increased, while BSYS treatment significantly reversed this effect and modulated the levels of C/EBP *α*, PU.1, and SOCS1 (target genes of miR-124 and miR-155). Therefore, the neuroprotective effect of BSYS against MS/EAE was related to promoting microglia toward M2 polarization, which may be correlated with changes in miR-124 and miR-155 in vivo.

## 1. Introduction

Multiple sclerosis (MS) is a chronic inflammatory disease of the central nervous system (CNS) characterized by demyelination [1], neurodegeneration, and sensitivity to oxidative stress [2, 3]. The disease usually occurs in young adults, and its main clinical manifestations include limb weakness, sensory abnormality, visual impairment, and ataxia [4]. Approximately 2.5 million people have MS worldwide [5]. The neuroimmune inflammatory response plays a crucial role in the pathogenesis of MS. Therefore, anti-inflammatory strategies have attracted much attention in MS treatment.

Microglia are macrophages that reside in the brain and spinal cord and participate in the inflammatory process of the CNS [6]. Under inflammatory conditions in the brain, such as Parkinson's disease, multiple system atrophy, and MS, microglia may be rapidly activated and are polarized into M1 and M2 phenotypes [7]. M1 type microglia play a strong phagocytic role and release proinflammatory factors such as tumor necrosis factor- (TNF-) *α*, interleukin- (IL-) 1*β*, and IL-6, which aggravate the inflammatory reaction, resulting in nerve injury [8]. M2 microglia not only release anti-inflammatory factors, including IL-10, to reduce the level of inflammation but also secrete transforming growth factor- (TGF-) *β* to promote tissue repair and myelin regeneration [9]. Microglia polarize to the proinflammatory M1 type during MS onset, leading to an imbalance in M1/M2 in vivo, forming a proinflammatory microenvironment in the CNS and causing damage to the myelin sheath. Therefore, developing new drugs that promote M2 polarization may provide an effective treatment strategy in neurodegenerative diseases [10].

As crucial modulators of gene expression, microRNAs (miRs) have been widely studied in recent years; they are involved in posttranscriptional regulation and play an important role in modifying microglial polarization. Many studies have suggested that the biological processes of microglial polarization modulated by miRs are correlated with the immune inflammatory response of MS/EAE. miR-124 expression in the CNS of EAE was decreased, while that of miR-155 was increased [11, 12]. However, miR-124 upregulation [13] or miR-155 downregulation [14, 15] in vivo significantly alleviates the severity of EAE. Additionally, miR-124 and miR-155 are considered important regulators to balance the ratio of M1/M2 [16, 17], and a previous study demonstrated that miR-124 alleviates EAE not only by promoting microglial quiescence but also by skewing microglial polarization from the M1 phenotype to the M2 phenotype via targeting C/EBP*α*-PU.1 [18]. Furthermore, miR-155 is important for microglial polarization; it targets suppressor of cytokine signaling 1 (SOCS1) to promote macrophage/microglia polarization toward the M1 phenotype [19], while miR-155 deficiency leads to a shift from M1 to M2 [20]. Consequently, elevating the expression of miR-124 and decreasing the expression of miR-155 might be a promising therapeutic approach for EAE treatment by promoting microglial phenotypic transformation toward M2.

Growing evidence has confirmed that herbs in traditional Chinese medicine (TCM) are effective in treating neurodegenerative diseases of the CNS, including Alzheimer's disease (AD) [21, 22], amyotrophic lateral sclerosis (ALS) [23], and MS. Previous studies have shown that numerous prescriptions and herbal extracts of TCM have protective effects against diseases by modulating various miRs [24–27]. Thus, it is reasonable to explore new therapeutic strategies based on TCM to treat MS. Bu Shen Yi Sui capsule (BSYS) was modified from the Liu Wei Di Huang pill, a well-known traditional Chinese formula, which has been used for 10 years in MS clinical treatment at Tiantan Hospital (Beijing, China). Our previous studies found that BSYS could ameliorate demyelination and axon injury by regulating Th17/Treg cells [28] and promoting oligodendrocyte progenitor cell (OPC) maturation [29, 30]. This study is aimed at evaluating the neuroprotective effects of BSYS on EAE mice and potential mechanisms of microglial polarization regulated by BSYS.

## 2. Materials and Methods

### 2.1. Drug Preparations

BSYS comprised the following Chinese herbs: Dihuang (Rehmanniae Radix), Shu dihuang (Rehmanniae Radix Praeparata), Heshouwu (Polygoni Multiflori Radix), Dahuang, (Rhei Radix et Rhizoma), Yimucao (Leonuri Herba), Zhebeimu (Fritillariae Thunbergii Bulbus), Shuizhi (Hirudo), Quanxie (Scorpio), Tianma (Gastrodiae Rhizoma), and Lianqiao (Forsythiae Fructus). BSYS was produced by Asia-East Biopharmaceutical Co., Ltd. (Beijing, China). A detailed description of the BSYS preparation and quality control procedures has been published elsewhere [31] [32]. Fingolimod (FTY720) was used as a positive control drug and was purchased from Novartis (Basel, Switzerland).

### 2.2. Animals

Healthy female C57BL/6J mice at 6–8 weeks of age (weighing between 15 and 17 g) were purchased from Vital River (Beijing, China) and were housed under specific pathogen-free (SPF) conditions in a 12/12 h light/dark cycle room with controlled temperature (22 ± 3°C) and humidity (40–50%) at the Experimental Animal Center of Capital Medical University. All the mouse experiments were performed under the approval of the Animal Experiments and Experimental Animal Welfare Committee of Capital Medical University (permit number: AEEI-2018-137).

### 2.3. Model Establishment and Experimental Treatment

Five milligrams of myelin oligodendrocyte glycoprotein (MOG)_35–55_ peptide, dissolved in normal saline (10 ml), was emulsified 1 : 1 in complete Freund's adjuvant supplemented with 0.3 mg of *Mycobacterium tuberculosis* H37Ra (BD Biosciences, San Diego, USA). Next, 0.2 ml of emulsion containing 50 *μ*g of MOG_35–55_ was administered subcutaneously into 4 different locations on the backs of female C57BL/6J mice. On Day 0 and Day 2 postimmunization (dpi), C57BL/6 mice were injected intraperitoneally with 500 ng of pertussis toxin (Sigma-Aldrich, St. Louis, USA).

The mice were randomly divided into four groups: (1) normal control group (NC, *n* = 10), (2) EAE model group (EAE, *n* = 10), (3) 0.3 mg/kg FTY720 treatment group [33, 34] (FTY720, *n* = 10), and 3.02 g/kg BSYS treatment group (BSYS, *n* = 10). Additionally, the clinical equivalent daily dose of BSYS in mice was 3.02 g/kg, which was the optimal dosage against EAE in our previous study [35]. The therapeutic group was administered the corresponding dose of BSYS or FTY720 daily by gavage, while the same volume of distilled water was used for the NC and EAE groups.

After the animal model was established, the clinical signs of EAE in mice were observed daily for 40 days based on the following grades. The score for tail deficiency was as follows: 0, normal; 1, partially paralyzed; and 2, completely paralyzed. Each hindlimb or forelimb was evaluated as follows [36, 37]: 0, normal; 1, weak or altered gait; 2, paresis; and 3, completely paralyzed limb. Therefore, according to this scoring system, a mouse with complete quadriplegia will receive 14 points, while death will be scored as 15 points.

### 2.4. CNS Histopathological Staining

At 40 dpi, the mice were deeply anesthetized, sacrificed, and perfused with phosphate-buffered saline (PBS) and 4% paraformaldehyde (PFA). Brain and spinal cord sections from the subventricular zone (SVZ), corpus callosum (CC), and lumbar enlargement (LE) in the mice of the four groups were immersed in 4% PFA for 24 h and then embedded in paraffin. Tissue sections (5 *μ*m) were subjected to hematoxylin and eosin (H&E) staining and Luxol Fast Blue (LFB) staining to assess the degree of inflammatory infiltration and demyelination. The inflammation level was assessed based on the following criteria [38]: 0, no inflammation; 1, the cells only infiltrated around the blood vessels and meninges rarely; 2, 1-10 cells infiltrated per area (mild cellular infiltration); 3, 11-100 cells infiltrated per area (moderate cellular infiltration); and 4, >100 cells infiltrated per area (serious cellular infiltration). As mentioned previously, the demyelination degree was calculated based on a 3-point scale as follows [39]: 0 = no demyelination area; 1 = a few foci of demyelination; 2 = a few areas of demyelination; and 3 = large areas of demyelination.

### 2.5. Transmission Electron Microscopy (TEM)

To evaluate the degree of demyelination, tissues were cut into approximately 1 × 1 × 3 mm^3^ pieces, placed in 2.5% glutaraldehyde for 2 h, and then rinsed with 0.1 M PB buffer three times. Next, the tissues were fixed with 1% osmium acid, dehydrated with alcohol, soaked for 20 min, embedded with embedding agent, and baked and soaked in pure embedding agent. The embedded samples were sliced using an ultrathin microtome and then stained and coated. The sections were then viewed under TEM (H-7700; Hitachi, Japan) at a magnification of ×5000. The G-ratio was defined as the ratio of the diameter of a given axon and myelinated fiber diameter [40], which were measured 50 times for each group using ImageJ (NIH, Bethesda, USA).

### 2.6. Immunofluorescence Staining

Brain and spinal cord sections from the SVZ and LE in the mice of the four groups were deparaffinized, boiled with citrate buffer at 95°C for 20 min, cooled to 30°C, and blocked with 1% bovine serum albumin (BSA) at 37°C for 1 h. The sections were then incubated overnight at 4°C with primary antibodies against Olig2 (1 : 400; AF2418; R&D Systems, Minneapolis, USA), CC-1 (1 : 200; OP80; Merck Chemicals, Darmstadt, Germany), iNOS (1 : 200; ab210823; Abcam, Cambridge, UK), Arginase 1 (Arg1; 1 : 200; ab91279; Abcam, Cambridge, UK), Iba1 (1 : 200; ab48004; Abcam, Cambridge, UK), followed by 1 h of incubation with FITC- (1 : 400-) and TRITC- (1 : 200-) conjugated secondary antibodies (Southern Biotech, Birmingham, USA) at 37°C. Nuclei were counterstained with DAPI (Southern Biotech, Birmingham, USA). We used a fluorescence microscope to capture the images and then analyzed them using ImageJ.

### 2.7. Enzyme-Linked Immunosorbent Assay (ELISA)

The brain and spinal cord samples were homogenized using a high-speed homogenizer and centrifuged at 5000 × g at 4°C for 10 min, and then, the supernatants were collected. The total protein concentration in the supernatants of each sample was calculated using a BCA protein assay kit (Applygen, Beijing, China). The expression levels of TNF-*α*, IL-1*β*, IL-6, and IL-10 were measured using ELISA kits (Neobioscience, Shenzhen, China) according to the manufacturer's protocol.

### 2.8. Quantitative Real-Time PCR (qRT-PCR) Analysis

For mRNA expression detection, qRT-PCR analysis was performed using the One-Step qPCR kit (Toyobo Life Science, Osaka, Japan) and the SYBR Green method in the CFX Connect Real-Time PCR Detection System (Bio-Rad, Hercules, USA). The primers for gene amplification were as follows:

major histocompatibility complex-II (MHC-II, FWD-GATGTGGAAGACCTGCG, REV-TGCATCTTCTGAGGGGTTTC), inducible nitric oxide synthase (iNOS, FWD-CTGTGAGACCTTTGATGTCCGAAG, REV-CTGGATGAGCCTATATTGCTGTGG), cluster of differentiation marker 86 (CD86, FWD-GCCACCCACAGGATCAATTATCCT, REV-AAAGAGAGAGGCTGTTGGAGATAC), Trem2 (FWD-TGGAACCGTCACCATCACTC, REV-TGGTCATCTAGAGGGTCCTCC), Arg1 (FWD-CTTGGCTTGCTTCGGAACTC, REV-GGAGAAGGCGTTTGCTTAGTTC), CD206 (FWD-AGTGATGGTTCTCCTGTTTCC, REV-GGTGTAGGCTCGGGTAGTAGT), C/EBP*α* (FWD-AGCTTACAACAGGCCAGGTTTC, REV-CGGCTGGCGACATACAGTAC), PU.1 (FWD-CCCGGATGTGCTTCCCTTAT, REV-TCCAAGCCATCAGCTTCTCC), SOCS1 (FWD-CACCTTCTTGGTGCGCG, REV-AAGCCATCTTCACGCTGAGC), and *β*-actin (FWD-ATATCGCTGCGCTGGTCGTC, REV-AGGATGGCGTGAGGGAGAGC).

Real-time PCR was performed using the CFX Connect Real-Time System and the following reaction conditions: incubation at 95°C for 60 s, followed by 40 cycles of 95°C for 15 s (denaturation), 60°C for 15 s (annealing), and 72°C for 45 s (extension). The mRNA expression data were normalized to *β*-actin expression, while the level of miR-124/miR-155 was normalized to the U6 level. The data were analyzed using the relative quantification (2^-*ΔΔ*Ct^) method.

### 2.9. hFluorescence In Situ Hybridization

Fluorescence in situ hybridization (FISH) was performed on PFA-fixed, paraffin-embedded sections to detect miR-124 and miR-155 in brain and spinal cord tissue. The staining was performed according to established protocols. Briefly, 4% PFA-fixed paraffin-embedded sections were preheated for 2 h at 62°C. Xylene was used to remove paraffin from the tissue. The sections were incubated in decreasing concentrations of ethanol and then incubated with Proteinase K solution for 20-30 min at 37°C. After dehydration, biotin-labeled miR probes were added to the hybridization solution and incubated at 37°C overnight. The sections were counterstained with DAPI at room temperature (RT) for 8 min (in the dark). Finally, we used a fluorescence microscope to observe the slices.

### 2.10. Isolation and Identification of Exosomes from Mouse Serum

Exosomes were isolated from the serum of mice using an exoRNeasy Serum Mid Kit (Qiagen, Hilden, Germany) following the manufacturer's protocol. Measurements of the average diameter and size distribution of serum exosomes were performed using nanoparticle tracking analysis (NTA), and morphological assessment was performed using TEM as described in our previous study. Biomarkers of exosomes, such as CD63, HSP70, and TSG101, were examined in exosome-depleted media and exosomes isolated from serum using western blot analysis.

### 2.11. Western Blot Analysis

Tissues were lysed in RIPA buffer containing a protease inhibitor cocktail (Beyotime Institute of Biotechnology, Haimen, China). Total protein isolated from the myocardium was separated by SDS-PAGE and transferred to polyvinylidene difluoride (PVDF) membranes. The membranes were blocked in Tris-buffered saline with 0.1% Tween (TBST) containing 5% nonfat dry milk for 1 h at RT and then incubated in universal antibody diluent (New Cell & Molecular Biotech, Suzhou, China) using the appropriate primary antibody overnight at 4°C. The primary antibodies used in this experiment were specific to the following antigens: iNOS (1 : 1000; ab210823; Abcam, Cambridge, UK), Arg1 (1 : 1000; ab91279; Abcam, Cambridge, UK), C/EBP*α* (1 : 1000; 18311-1-AP; Proteintech, Chicago, USA), PU.1 (1 : 2000; ab88082; Abcam, Cambridge, UK), SOCS1 (1 : 1000; YT4362; Immunoway, Newark, USA), CD63 (1 : 1000; ab213090; Abcam, Cambridge, UK), HSP70 (1 : 1000; ab2787; Abcam, Cambridge, UK), and TSG101 (1 : 1000; ab125011; Abcam, Cambridge, UK). The membranes were incubated with HRP-conjugated secondary antibodies after washing in 5% TBST. Finally, the protein bands were detected using a gel chemiluminescence imaging analysis system and the Immobilon Western Chemiluminescent HRP Substrate reagent (Millipore, Billerica, USA) and then were analyzed using ImageJ.

### 2.12. Statistical Analysis

Statistical analyses were performed using GraphPad Prism (version 8.01; San Diego, USA). H&E and LFB score statistics were analyzed using the Kruskal-Wallis test followed by Dunn's test. The EAE clinical scores and body weight were analyzed using two-way ANOVA with the Bonferroni posttest. Other analyses of three or more groups were performed using one-way ANOVA and the Bonferroni posttest. Values of *P* < 0.05 were considered statistically significant.

## 3. Results

### 3.1. BSYS Alleviates the Clinical Severity of EAE Mice

**Figure 1:**
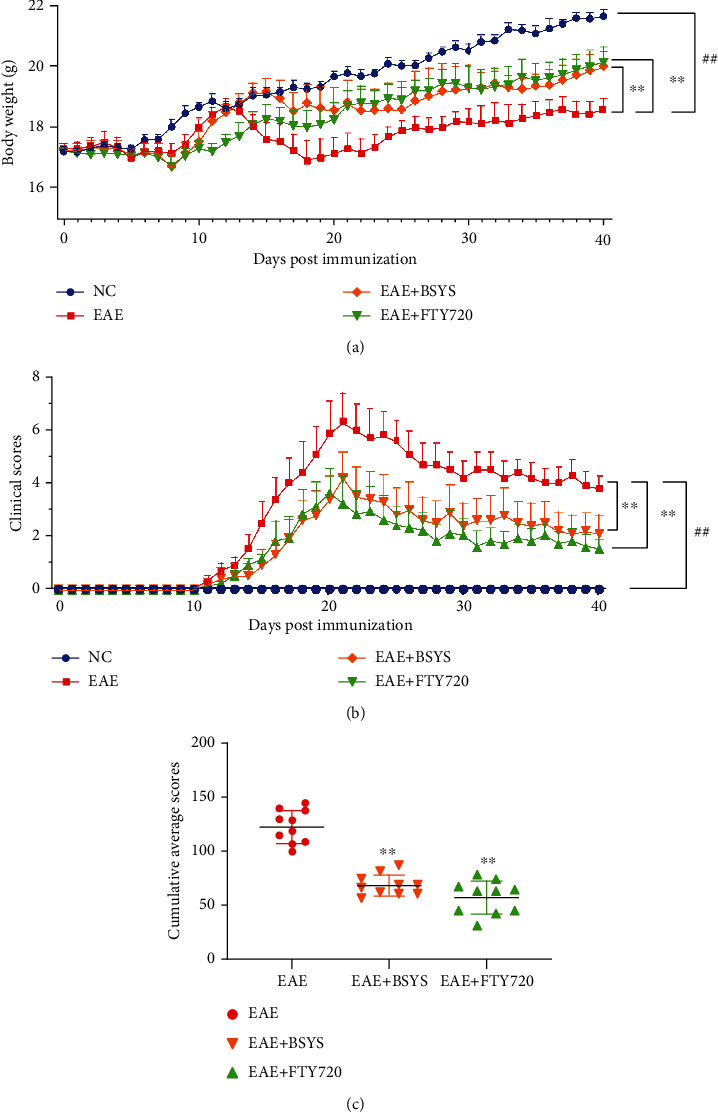
BSYS improved the body weight and ameliorated clinical symptoms of EAE mice: (a) changes in body weight of mice in each group (*n* = 10); (b) changes in the time of mean clinical score of mice in each group (*n* = 10); (c) cumulative clinical scores of different groups. The data are expressed as mean ± SEM, compared with the NC group, ^##^*P* < 0.01; compared with the EAE group, ^∗∗^*P* < 0.01.

To evaluate the therapeutic effect of BSYS on EAE mice, the body weight and clinical score were tested daily for 40 days. The body weights of EAE mice were markedly decreased from 13 to 40 dpi compared with those of NC mice; however, this tendency was alleviated in the FTY720 and BSYS groups (Figure 1(a)). No neurological symptoms were observed in the NC group during the entire 40 days. Compared with the NC group, the EAE group began to show neurological deficit symptoms at 11 dpi, and the clinical score of EAE mice increased rapidly, peaking at 21 dpi. From 11 dpi to 40 dpi, the increase in the clinical score in the FTY720 and BSYS groups was markedly lower than that in the EAE group (Figure 1(b)). The cumulative average scores of mice in the FTY720 and BSYS groups were also markedly decreased compared with those of the EAE group (Figure 1(c)).

### 3.2. BSYS Suppresses Inflammatory Cell Infiltration and Attenuated Demyelination

**Figure 2:**
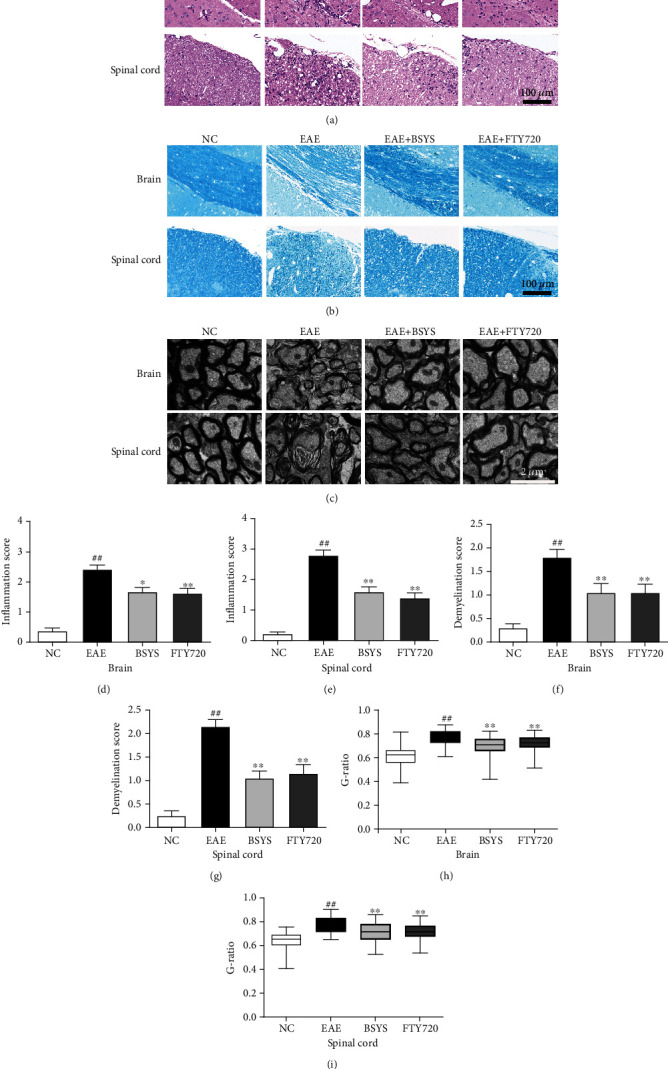
BSYS suppressed inflammatory cell infiltration and promoted myelination in EAE mice. (a) Inflammatory infiltrates of the SVZ and LE of mice in each group were observed by H&E staining. Scale bars: 100 *μ*m. (b) Demyelination of the SVZ and LE of mice in each group was observed by LFB staining. Scale bars: 100 *μ*m. (c) Myelin ultrastructure of the SVZ and LE of mice in each group was observed by TEM. Scale bars: 2 *μ*m. (d, e) Histological quantification of H&E-stained sections in each group. (f, g) Histological quantification of LFB-stained sections in each group. (h, i) G-ratio of axons in spinal cords from mice in each group at day 40. Data are presented as means ± SD; compared with the NC group, ^##^*P* < 0.01; compared with the EAE group, ^∗^*P* < 0.05, ^∗∗^*P* < 0.01.

To further study the protective effect of BSYS on the degree of inflammation and demyelination, tissues in the brain (SVZ, CC) and spinal cord (LE) were observed by H&E and LFB staining. The EAE group showed a larger amount of inflammatory cell infiltration than the NC group (Figure 2(a)). Treatment with either BSYS or FTY720 alleviated the pathological severity. Additionally, large demyelination plaques were observed in the LE and CC of untreated EAE mice, while FTY720 and BSYS reduced the demyelination area in EAE mice (Figure 2(b)). Additionally, we observed the ultrastructure of the myelin sheath by TEM. The myelin sheath of the NC group was closely arranged, the structure was intact, and the morphology of mitochondria was normal. By contrast, untreated EAE mice showed a loose lamellar myelin structure, axonal edema, and nerve fiber disintegration. The ratio of the axon diameter to myelinated axon diameter (G-ratio) was used to quantify the degree of demyelination. Compared with that of the untreated group, the G-ratio of EAE mice treated with BSYS and FTY720 markedly decreased, and the integrity of the myelin sheath was protected (Figure 2(c)).

### 3.3. BSYS Promotes the Maturation of Oligodendrocytes in EAE Mice

**Figure 3:**
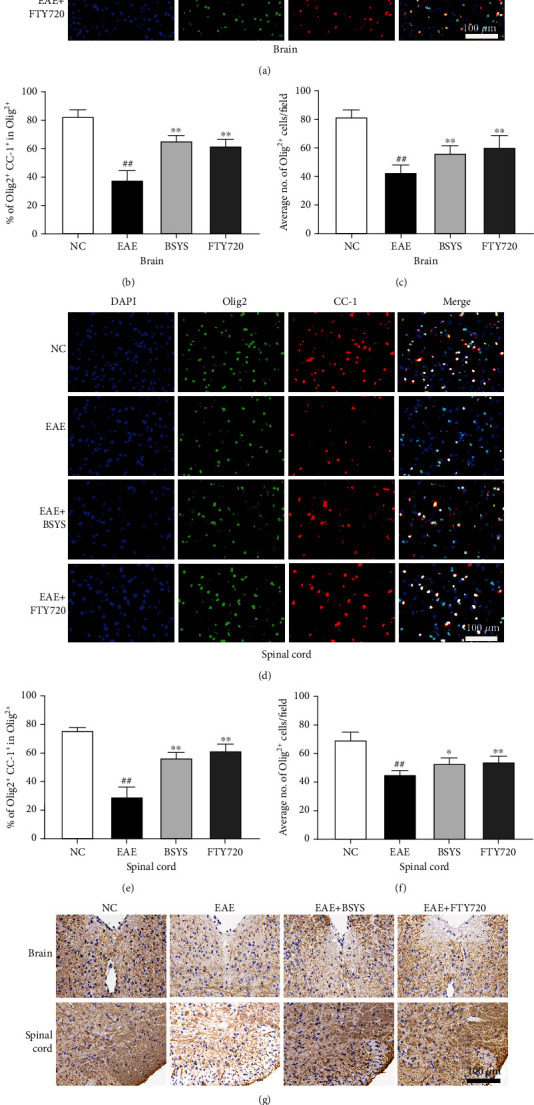
BSYS promoted the maturation of oligodendrocytes in EAE mice. (a) Immunofluorescence of SVZ from mice in each group using antibodies against Olig2 (green) and CC-1 (red). Scale bars: 100 *μ*m. (b) Quantification of Olig2+ cells density in SVZ. (c) Quantification of the percentage of CC-1+/Olig2+ cells in LE. (d) Immunofluorescence of LE using antibodies against Olig2 and CC-1. Scale bars: 100 *μ*m. (e) Quantification of Olig2+ cells density in LE. (f) Quantification of the percentage of CC-1+/Olig2+ cells in LE. (g) The expression of PLP in SVZ and LE was detected by immunohistochemistry. Scale bars: 100 *μ*m. (h, i) Percentage of MBP covered area in the SVZ and LE. Data are presented as means ± SD; compared with the NC group, ^##^*P* < 0.01; compared with the EAE group, ^∗^*P* < 0.05, ^∗∗^*P* < 0.01.

Considering the significant improvement in neurological results, we aimed to explore whether BSYS affects the maturation of oligodendrocytes and remyelination process, which exerts a crucial effect on the recovery of EAE. CC-1 is a mature oligodendrocyte marker [41]. Immunofluorescence showed that, in both the brain and spinal cord, the CC-1^+^/Olig2^+^ cell ratio in the BSYS-treated group was clearly higher than that in the EAE group (Figures 3(a)–3(f)).

Myelin proteolipid protein (PLP) is another marker of mature oligodendrocytes and one of the main membrane proteins that make up myelin [42]. We quantified PLP in the brain and spinal cord by IHC staining, revealing that the level of PLP in EAE mice was significantly reduced at 40 dpi. However, the PLP expression was increased in the FTY720- and BSYS-treated groups compared with that in the EAE group (Figures 3(g)–3(i)).

### 3.4. BSYS Treatment Promotes M2 Microglial Polarization in the Brain and Spinal Cord of EAE Mice

**Figure 4:**
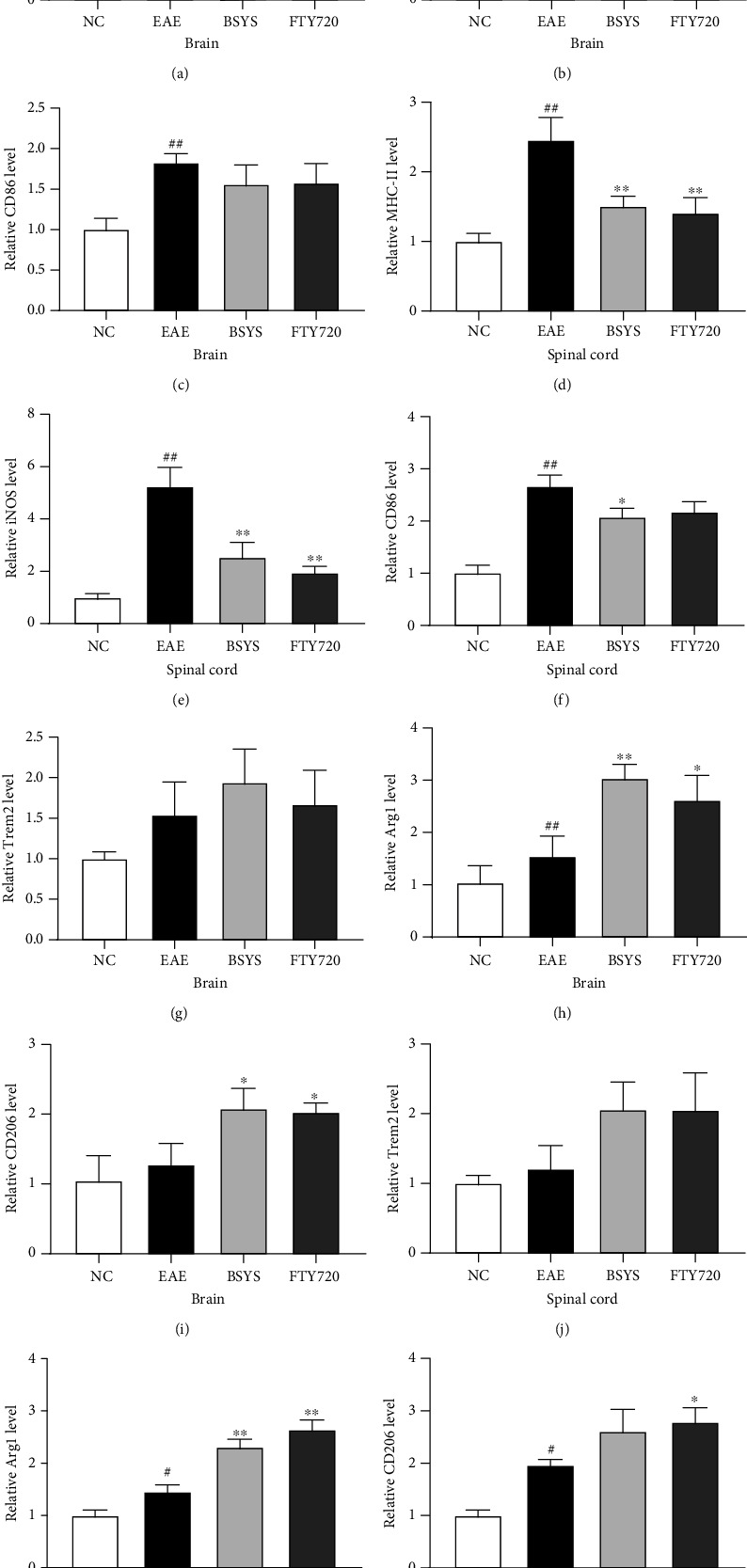
Effect of BSYS on microglial polarization markers. (a–f) M1 phenotype markers (MHC-II, iNOS, CD86) in brain and spinal cord were detected by qRT-PCR. (g–l) M2 phenotype markers (Trem2, Arg1, CD206) in brain and spinal cord were detected by qRT-PCR. Data are presented as means ± SD; compared with the NC group, ^#^*P* < 0.05, ^##^*P* < 0.01; compared with the EAE group, ^∗^*P* < 0.05, ^∗∗^*P* < 0.01.

To explore the role of BSYS in microglial polarization, the mRNA expression levels of M1 markers (MHC-II, iNOS, CD86) and M2 markers (Trem2, Arg1, CD206) were measured at 40 dpi in the mouse brain and spinal cord. Compared with those in the NC group, the mRNA levels of M1 phenotype markers were elevated in the EAE group (Figures 4(a)–4(f)). However, BSYS and FTY720 treatment reduced the expression levels of M1-related markers; in particular, MHC-II and iNOS were significantly downregulated. By contrast, the levels of M2 polarization markers were increased in the BSYS-treated group compared with that in the EAE group, but only Arg1 upregulation was statistically significant in both the brain and spinal cord (Figures 4(g)–4(l)). These outcomes suggested that the antidemyelination effect of BSYS may be partly realized by suppressing M1 microglial polarization and reversing microglial polarization toward the M2 phenotype.

**Figure 5:**
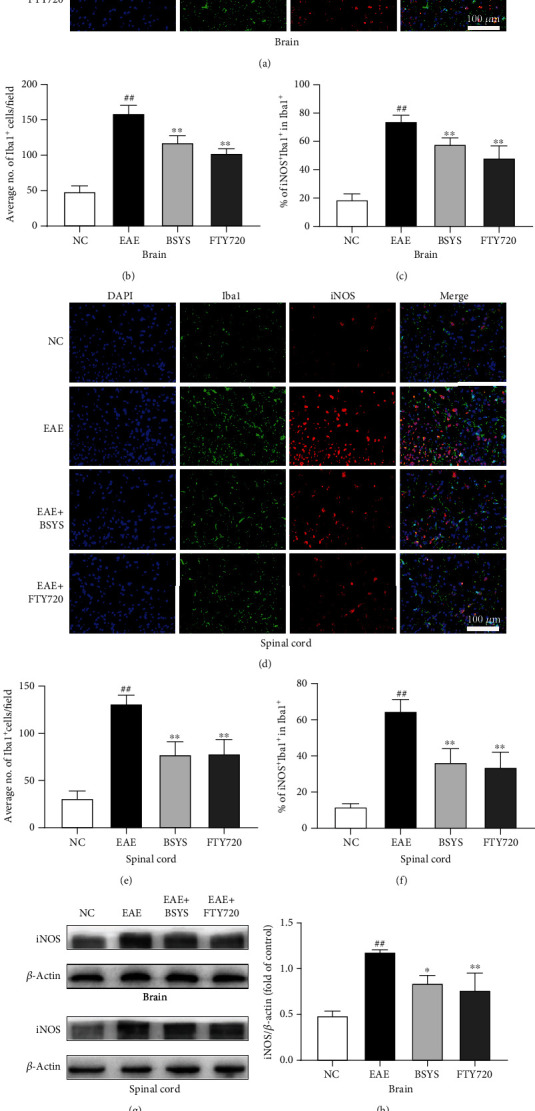
BSYS inhibited the polarization of microglia to M1 phenotype in EAE mice. (a, d) Immunofluorescence of SVZ and LE from mice in each group using antibodies against Iba1 (green) and iNOS (red). Scale bars: 100 *μ*m. (b, e) Quantification of the percentage of iNOS+/Iba1+ cells in SVZ and LE. (c, f) Quantification of Iba + cells density in SVZ and LE. (g) Representative western blot images of iNOS in brain and spinal cord tissues. (h, i) Quantitative data for iNOS expression in brain and spinal cord tissues. Data are presented as means ± SD; compared with the NC group, ^##^*P* < 0.01; compared with the EAE group, ^∗^*P* < 0.05, ^∗∗^*P* < 0.01.

**Figure 6:**
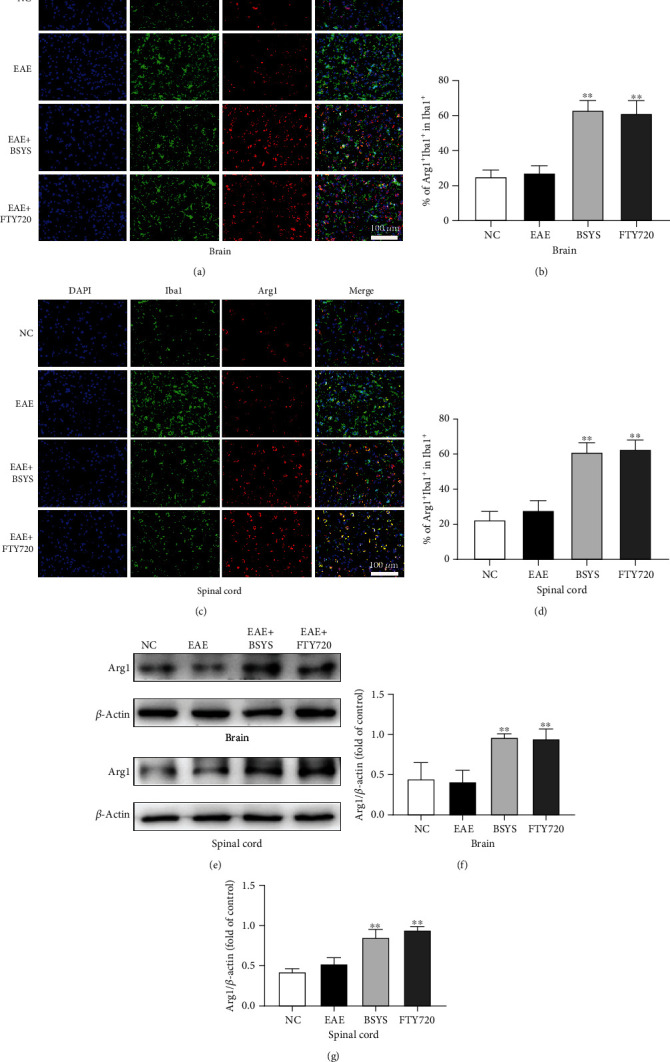
BSYS promoted the polarization of microglia to M2 phenotype in EAE mice. (a, c) Immunofluorescence of SVZ and LE from mice in each group using antibodies against Iba1 (green) and Arg1 (red). Scale bars: 100 *μ*m. (b, d) Quantification of the percentage of Arg1+/Iba1+ cells in SVZ and LE. (e) Representative western blot images of Arg1 in brain and spinal cord tissues. (f, g) Quantitative data for Arg1 expression in brain and spinal cord tissues. Data are presented as means ± SD; compared with the EAE group, ^∗∗^*P* < 0.01.

Next, microglial polarization was further investigated in the CNS by immunofluorescence. Brain and spinal cord sections were double-stained for Iba1 (microglial marker)/iNOS (M1 marker) or Iba1/Arg1 (M2 marker). EAE induced marked upregulation in the ratio of M1 microglia. However, BSYS and FTY720 treatment decreased the numbers of activated microglia (Iba1^+^ cells) (Figures 5(a), 5(b), 5(d), and 5(e)) and M1 microglia (Iba1^+^/iNOS^+^ cells) (Figures 5(a), 5(c), 5(d), and 5(f)) and increased the proportion of M2 microglia in the treatment groups compared with that in the EAE group (Figures 6(a)–6(d)). Next, western blot analysis showed that the iNOS expression was significantly increased in the EAE group compared with that in the NC group. However, BSYS and FTY720 reversed the upregulation of iNOS (Figures 5(g)–5(i)) and downregulation of Arg1 induced by EAE (Figures 6(e)–6(g)). These outcomes were consistent with the results of immunofluorescence staining.

**Figure 7:**
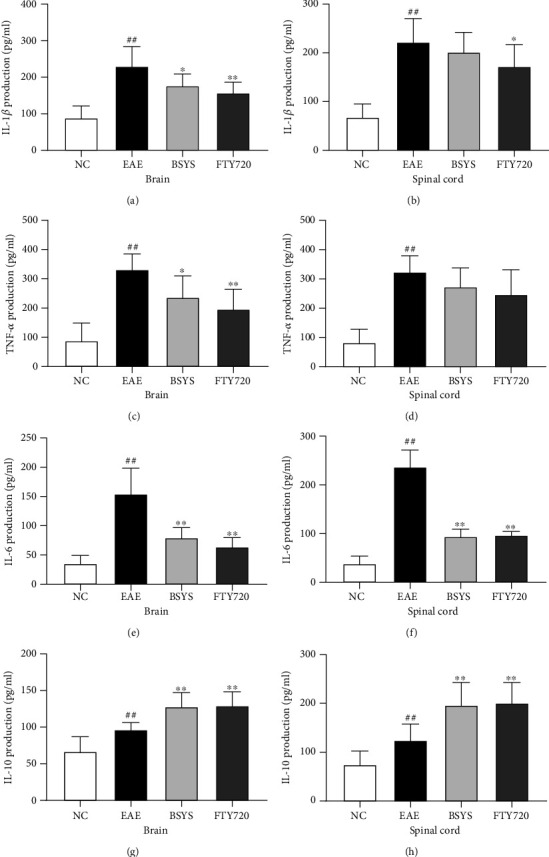
Effect of BSYS on cytokine levels of CNS in EAE mice. (a–f) The levels of proinflammatory cytokines (IL-1*β*, TNF-*α*, IL-6) in brain and spinal cord were detected by ELISA. (g–h) The levels of anti-inflammatory cytokine (Trem2, Arg1, CD206) in brain and spinal cord were detected by ELISA. Data are presented as means ± SD; compared with the NC group, ^##^*P* < 0.01; compared with the EAE group, ^∗^*P* < 0.05, ^∗∗^*P* < 0.01.

M1 and M2 microglia secrete pro- and anti-inflammatory cytokines, respectively, and ELISA was used to further evaluate the efficacy of BSYS in modulating microglial polarization. ELISA showed that the expression levels of the inflammatory cytokines IL-1*β*, IL-6, and TNF-*α* in the EAE group were higher than those in the NC group. The administration of FTY720 and BSYS suppressed the increase in proinflammatory cytokines in the CNS induced by EAE (Figures 7(a)–7(f)). However, compared with the EAE group, only the IL-6 reduction was statistically significant in both the brain and spinal cord in the BSYS treatment group. Moreover, the levels of IL-10 (Figures 7(g)–7(h)) secreted by M2 phenotype microglia were markedly increased in the BSYS group compared with those in the EAE group.

### 3.5. BSYS Regulates miR-124 and miR-155 in CNS

**Figure 8:**
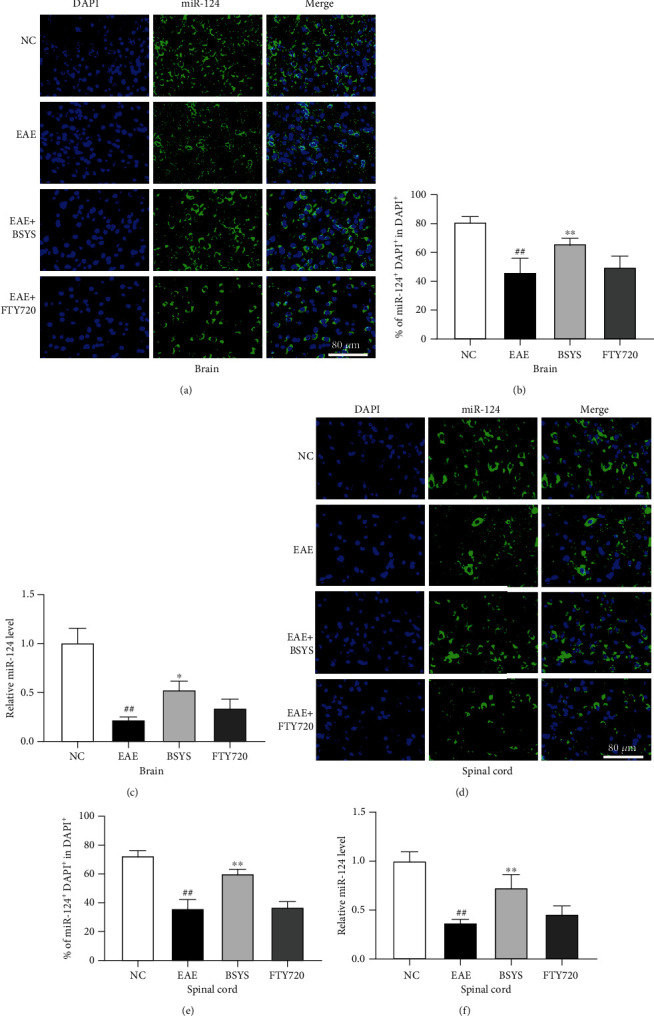
BSYS upregulated the expression of miR-124 in EAE mice. (a, d) miR-124 was labeled in SVZ and LE by fluorescence in situ hybridization. Scale bars: 80 *μ*m. (b, e) Quantification of miR-124 + density in SVZ and LE. (c, f) The expression levels of miR-124 in brain and spinal cord from mice of each group were detected by qRT-PCR. Data are presented as means ± SD; compared with the NC group, ^##^*P* < 0.01; compared with the EAE group, ^∗^*P* < 0.05, ^∗∗^*P* < 0.01.

**Figure 9:**
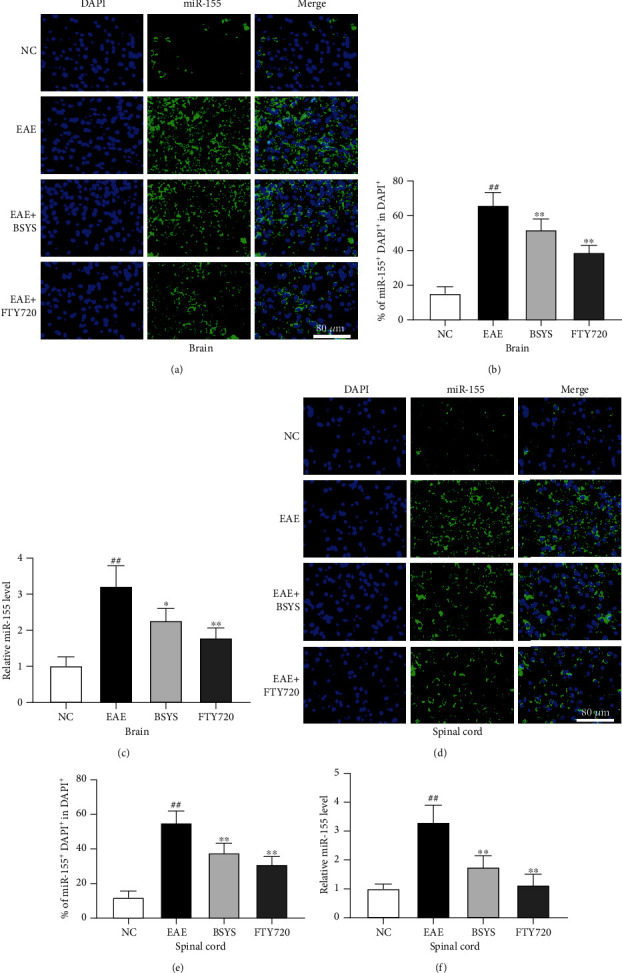
BSYS downregulated the expression of miR-155 in EAE mice. (a, d) miR-155 was labeled in SVZ and LE by fluorescence in situ hybridization. Scale bars: 80 *μ*m. (b, e) Quantification of miR-155 + density in SVZ and LE. (c, f) The expression levels of miR-155 in brain and spinal cord from mice of each group were detected by qRT-PCR. Data are presented as means ± SD; compared with the NC group, ^##^*P* < 0.01; compared with the EAE group, ^∗^*P* < 0.05, ^∗∗^*P* < 0.01.

Many studies have shown that miR-124 and miR-155 play anti-inflammatory and proinflammatory effects in neuroinflammation and are the key regulatory genes of microglial polarization. Upregulation of miR-124 and downregulation of miR-155 promote M2 polarization, thus reducing nerve injury and protecting neural function. qRT-PCR showed that the EAE model significantly increased the miR-155 expression in the brain and spinal cord compared with that in the NC group (Figures 8(c) and 8(f)), while the miR-124 level was downregulated (Figures 9(c) and 9(f)). Treatment of EAE mice with either BSYS or FTY720 led to a significant decrease in the relative miR-155 expression in the brain and spinal cord. Additionally, BSYS increased the miR-124 expression. We also detected miRs in the SVZ and LE by fluorescence in situ hybridization, and the results were consistent with those of qRT-PCR (Figures 8(a), 8(b), 8(d), and 8(e); Figures 9(a), 9(b), 9(d), and 9(e)), indicating that BSYS regulates miR-124 and miR-155 in the CNS to play a potential role in promoting M2 polarization of microglia.

### 3.6. BSYS Increases miR-124 and Suppresses miR-155 in Peripheral Serum Exosomes

**Figure 10:**
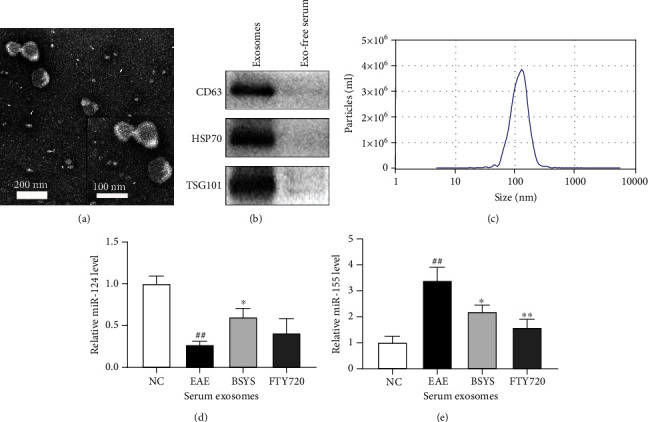
BSYS regulated the expression of serum exosomal miR-124 and miR-155 in EAE mice. (a) Exosomes derived from mice serum were observed by transmission electron microscopy. (b) Exosome markers (CD63, HSP70, and TGS101) were detected by western blot. (c) Particle sizes of serum exosomes were measured by NTA. (d, e) The expression levels of miR-124 and miR-155 in serum exosomes from mice of each group were detected by qRT-PCR. Data are presented as means ± SD; compared with the NC group, ^##^*P* < 0.01; compared with the EAE group, ^∗^*P* < 0.05, ^∗∗^*P* < 0.01.

In recent years, exosomes in peripheral circulation were demonstrated to likely serve as neuroinflammatory mediators [43], and the expression of some miRs in plasma exosomes is abnormal in the progression of MS/EAE disease [44, 45]. Therefore, at 40 dpi, we used an exoEasy Kit to collect serum exosomes from mice from each group and identified the morphology and size of exosomes by TEM and NTA. The collected vesicles showed typical cup-shaped morphology, and the diameter of the particles was 80~200 nm (Figures 10(a) and 10(c)). Exosomal markers, such as CD63, HSP70, and TSG101, were detected by western blotting and were enriched in isolated exosomes from serum (Figure 10(b)). Furthermore, we isolated exosomal RNA and analyzed the miR-124 and miR-155 expression in the serum exosomes of mice in each group by qRT-PCR. Compared with the serum exosomal miR-155 level in NC mice, that in EAE mice markedly increased, while the content of miR-124 decreased. BSYS treatment increased the miR-124 level and reduced the miR-155 level in the serum exosomes of EAE mice (Figures 10(d) and 10(e)). The results are consistent with the changes in the CNS tissues.

### 3.7. Target Genes of miR-124 and miR-155 Related to M2 Microglial Polarization Are Modulated by BSYS

**Figure 11:**
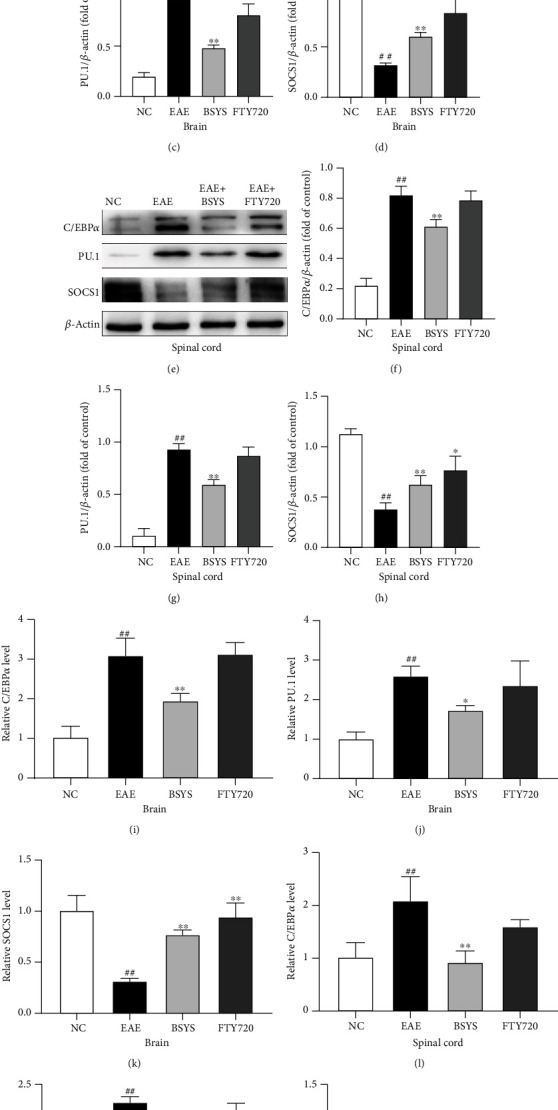
BSYS regulated the protein expressions of CEBP/*α*, PU.1, and SOCS1. (a, e) Representative images of western blot of CEBP/*α*, PU.1, and SOCS1 in the brain and spinal cord in EAE mice. (b–d, f–h) Quantitative analysis of CEBP/*α*, PU.1, SOCS1, and *β*-actin was used as the internal standard. (i–n) mRNA levels of CEBP/*α*, PU.1, and SOCS1 in the brain and spinal cord were detected by qRT-PCR. Data are presented as means ± SD; compared with the NC group, ^**##**^*P* < 0.01; compared with the EAE group, ^∗^*P* < 0.05, ^∗∗^*P* < 0.01.

C/EBP*α* and PU.1 are downstream target genes of miR-124, and SOCS1 is a target gene of miR-155. Many studies have shown that changes in these genes can regulate microglial polarization and affect the progression of MS/EAE. Therefore, we examined these target genes by qRT-PCR and western blot analysis. The expression levels of C/EBP*α* and PU.1 were upregulated in the brain and spinal cord in the EAE group compared with those in the NC group at 40 dpi. However, compared with their levels in the EAE group, these proteins were markedly downregulated after BSYS treatment. By contrast, the expression level of SOCS1 was decreased in the EAE group compared with that in the NC group, and treatment with FTY720 and BSYS induced its expression compared with that in the EAE group at 40 dpi (Figures 11(a)–11(n)).

## 4. Discussion

MS is an autoimmune disease of the CNS characterized by inflammatory cell infiltration and demyelination with more than two million cases worldwide [46]. Currently, immunosuppressants (e.g., glucocorticoid) and immunomodulators (e.g., FTY720) are the main drugs for MS treatment; however, they have significant risks in terms of safety and side effects [47, 48]. Many studies have focused on developing new drugs that inhibit demyelination to assist functional and pathological recovery in EAE. In our study, we assessed a TCM called BSYS, a formula that has shown therapeutic efficacy in MS/EAE and determined the potential mechanism of its neuroprotective effects. The body weight and clinical scores showed that BSYS treatment significantly improved neurologic recovery in EAE. Moreover, H&E and LFB staining showed that BSYS prevented inflammatory infiltration and demyelination in the brain and spinal cord. Additionally, the structure of the myelin sheath was protected in the lesion site after BSYS treatment. Furthermore, the number of cells that were CC-1^+^/Olig2^+^, markers of mature oligodendrocytes, was increased by BSYS treatment. PLP is the most abundant protein in the myelin sheath of the CNS. The PLP content is a quantitative indicator of myelin membrane integrity, and myelin also depends on its specific lipid content. Therefore, we speculated that BSYS affects PLP formation to reduce damage to the myelin sheath, which is supported by the expression levels of PLP observed in the brain and spinal cord. These results indicate that BSYS improves neurologic recovery in EAE mice by promoting oligodendrocyte maturation.

Previous studies clearly demonstrated that microglia regulate the microenvironment of the CNS and affect the process of remyelination [49]. In MS, microglia are activated and express signaling molecules and cytokines, leading to secondary nerve injury. Thus, inhibiting the overactivation of microglia and reducing the secretion of neurotoxic cytokines represent a new approach for MS treatment. Accumulating evidence has shown that microglial M1/M2 polarization plays a crucial role in neuroinflammation during EAE progression [50]. M1 microglia prevent oligodendrocyte maturation [51], while M2 microglia drive the differentiation of oligodendrocytes in neurodegenerative diseases [52]. Specifically, M1 microglia produce high levels of oxide metabolites, leading to CNS dysfunction. During neuroinflammation, many related receptors and enzymes in the M1 phenotype are upregulated to increase crosstalk and regulation of the CNS immune microenvironment, and MHC-II, iNOS, and CD86 are considered crucial markers [53]. The mRNA levels of these M1 phenotype markers (MHC-II, iNOS, CD86) increased in EAE mice. However, their expression was decreased in the BSYS group compared with that in the EAE group. However, the M2 phenotype secretes cytokines and expresses receptors related to anti-inflammation to induce tissue repair of the CNS, as demonstrated by the expression of triggering receptor expressed on myeloid cells 2 (Trem2), Arg1, and CD206 [54]. In our study, the mRNA expression of these genes was increased after BSYS treatment. Arg1 and iNOS share the same substrate; thus, only one of them is preferentially synthesized in microglia, making iNOS/Arg1 a suitable marker for microglial polarization. Indeed, consistent with our study, qRT-PCR showed that the most significant differences in M1 and M2 polarization markers between the EAE and treatment groups were in iNOS and Arg1, respectively. Thus, we used iNOS and Arg1 as markers to identify M1- and M2-polarized microglia in the CNS by immunofluorescence. Additionally, Iba1 is a microglia/macrophage-specific calcium-binding protein with actin-bundling activity that is widely used to identify activated microglia. FTY720 and BSYS treatment led to markedly increased expression of Arg1 (M2 marker) in Iba1^+^ cells, while iNOS (M1 marker) expression was decreased dramatically in EAE mice. Furthermore, M1 microglia secrete proinflammatory cytokines, while M2 microglia secrete cytokines that decrease inflammation [55]. Microglia activated in the EAE group were switched to the M1 phenotype and produced more proinflammatory cytokines to prevent remyelination. However, treatment with BSYS reduced the expression of IL-1*β*, IL-6, and TNF-*α* and increased the expression of IL-10, highlighting the neuroprotective effects of BSYS.

miRs are small non-protein-coding RNA molecules with a length of 18-25 nucleotides that play an important role in modulating gene expression and regulating diverse biological events [56]. In the past few years, miRs have been deemed potential regulators of microglial polarization during MS/EAE progression [18, 57]. Many studies have indicated that miRs alter the direction of microglial polarization, while the miRs expression level is different between the M1 and M2 phenotypes. In particular, miR-155 and miR-124 are representatives of the pro- and anti-inflammatory miRs, respectively, which are considered crucial to balance the polarization of M1 and M2 microglia [58].

Initially, miR-124 was assumed to be an anti-inflammatory miR [59] that contributes to suppressing microglial activation because the highest levels of miR-124 were detected in deactivated resident microglia, while the miR-124 levels were significantly decreased after stimulation with IFN-*γ* and LPS, which is known to potentiate the M1 phenotype. However, studies of miR-124-transfected microglia revealed that miR-124 decreased the expression of proteins associated with the M1 phenotype while increasing the expression of M2-associated markers. This result suggests that miR-124 not only deactivates microglia but also skews their polarization from the M1 phenotype toward the M2 phenotype [18]. Furthermore, during the acute phase of EAE, the increased levels of M1 markers in microglia occurred at the same time as the decrease in miR-124 expression in the CNS. By contrast, high expression of miR-124 and low expression of M1 markers were simultaneously observed in the CNS of normal mice or mice in the recovery phase of EAE [13, 60]. C/EBP*α* and PU.1 are downstream targets of miR-124, and C/EBP*α* is one of the C/EBP transcription factor family members that is widely expressed and regulates various cellular and physiological processes, including energy metabolism, immunity, and inflammation [61]. PU.1 serves as a critical regulator in the immune system. It exerts an important effect on regulating genes relevant to specialized functions of microglia [62, 63]. miR-124 controls multiple markers of microglial polarization by directly inhibiting C/EBP-*α* and its downstream transcription factor PU.1 [64]. miR-155, broadly considered a proinflammatory miR, contributes to microglia-mediated neurotoxicity, which is largely related to the M1 phenotype [65]. miR-155 was shown to be increased significantly in the MS/EAE brain and spinal cord [66]. It targets anti-inflammatory proteins in microglia, such as SOCS1, resulting in elevated expression levels of iNOS, IL-6, and TNF-*α*. Furthermore, SOCS1 induces differentiation from the M1 to M2 state and increased SOCS1 in the M2 phenotype, which plays an important role in sustaining the anti-inflammatory function [67]. Moreover, miR-155 can target M2-associated genes, and inhibition of miR-155 promotes the expression of M2 markers [68], such as Arg1, Ym1, and Fizz1. The above evidence suggests that the regulation of miR-124 and miR-155 in EAE has an important effect on the polarization of microglia, and the polarization of M1/M2 may also be accompanied by the changes of miR-124 and miR-155. In our study, the expression of miR-155 in the brain and spinal cord of EAE mice increased significantly because of autoimmune inflammation injury, while the expression of miR-124 decreased significantly compared with that in NC mice. However, after BSYS treatment, fluorescence in situ hybridization and qRT-PCR showed that the levels of miR-124 and miR-155 were reversed. These results indicate that microglial polarization to M2 caused by BSYS is related to changes in the miR expression.

Exosomes are secreted by various cells and carry cargos, including proteins, lipids, and noncoding RNAs [69]. After release, exosomes are transferred to specific target cells to exert multiple effects [70]. A recent study showed that peripheral circulating exosomes contribute to neuroinflammation under systemic inflammation conditions [43]. Increased expression of inflammatory miRs in serum-derived exosomes may play a role in regulating the CNS immune response. Furthermore, the expression of miRs in serum exosomes was dysregulated at the onset and peak of MS/EAE [71]. Thus, circulating exosomal miRs represent promising candidate biomarkers for MS/EAE and could reflect the status and therapeutic efficacy of the disease [44]. Our study found that the expression of serum exosomal miR-124 and miR-155 in EAE mice was similar to that in brain and spinal cord tissues; the former was downregulated, and the latter was upregulated. Furthermore, BSYS treatment reversed this trend, which was also consistent with the differences in the CNS tissues of mice in each group. Therefore, we confirmed the role of BSYS in regulating miRs at both the CNS and peripheral levels. Additionally, we analyzed the downstream target genes C/EBP *α*, PU.1, and SOCS1 of miR-124 and miR-155 in the CNS by western blotting and qRT-PCR. After BSYS treatment, the M1 phenotype-related SOCS1 was significantly suppressed, while the transcription factors C/EBP*α* and PU.1, which promote M2 polarization, were activated. These findings further suggest that the potential mechanism of BSYS is via promoting M2 polarization in the CNS of EAE mice, which may be related to the changes of miR-124 and miR-155 in vivo. However, all of our results were derived from in vivo studies. The role of miR-124 and miR-155 in BSYS-mediated microglia M2 polarization has not been fully elucidated, warranting further exploration. In future studies, we will inhibit and overexpress miR-124 and miR-155 in EAE mice to further study the mechanism of BSYS-mediated neuroprotection. Additionally, the specific mechanism of M2 polarization regulated by BSYS must also be proven in cell experiments, we will further verify these conclusions in vitro.

In summary, our study suggests that BSYS alleviates the inflammatory response, which suppresses demyelination by promoting M2 polarization of microglia to regulate the secretion of pro/anti-inflammatory factors and ameliorate neurological function. The effect of BSYS on skewing M2 polarization may correlate with the changes of miR-124 and miR-155 in vivo. Therefore, BSYS is a promising therapeutic agent to suppress neuroinflammation and improve remyelination.

## Data Availability

The data that support the findings of this study are available from the corresponding author upon reasonable request.
